# Machine learning and statistical approaches for classification of risk of coronary artery disease using plasma cytokines

**DOI:** 10.1186/s13040-021-00260-z

**Published:** 2021-04-15

**Authors:** Seema Singh Saharan, Pankaj Nagar, Kate Townsend Creasy, Eveline O. Stock, James Feng, Mary J. Malloy, John P. Kane

**Affiliations:** 1 Department of Statistics, University of Rajasthan, Jaipur, India; 2 Voluntary Data Scientist UCSF Kane Lab, San Francisco, USA; 3 UC Berkeley Extension, Berkeley, USA; 4 Department of Medicine, Cardiovascular Research Institute, University of California, San Francisco, USA; 5 Departments of Medicine and Pediatrics, Cardiovascular Research Institute, University of California, San Francisco, USA; 6 Department of Medicine, Department of Biochemistry and Biophysics, Cardiovascular Research Institute, University of California, San Francisco, USA

**Keywords:** CAD, ML, k-NN, Random Forest, Distance metrics, k-fold cross validation, Classification, AUROC, Confidence interval, Plasma cytokines, ROSE

## Abstract

**Background:**

As per the 2017 WHO fact sheet, Coronary Artery Disease (CAD) is the primary cause of death in the world, and accounts for 31% of total fatalities. The unprecedented 17.6 million deaths caused by CAD in 2016 underscores the urgent need to facilitate proactive and accelerated pre-emptive diagnosis. The innovative and emerging Machine Learning (ML) techniques can be leveraged to facilitate early detection of CAD which is a crucial factor in saving lives. The standard techniques like angiography, that provide reliable evidence are invasive and typically expensive and risky. In contrast, ML model generated diagnosis is non-invasive, fast, accurate and affordable. Therefore, ML algorithms can be used as a supplement or precursor to the conventional methods. This research demonstrates the implementation and comparative analysis of K Nearest Neighbor (k-NN) and Random Forest ML algorithms to achieve a targeted “At Risk” CAD classification using an emerging set of 35 cytokine biomarkers that are strongly indicative predictive variables that can be potential targets for therapy. To ensure better generalizability, mechanisms such as data balancing, repeated k-fold cross validation for hyperparameter tuning, were integrated within the models. To determine the separability efficacy of “At Risk” CAD versus Control achieved by the models, Area under Receiver Operating Characteristic (AUROC) metric is used which discriminates the classes by exhibiting tradeoff between the false positive and true positive rates.

**Results:**

A total of 2 classifiers were developed, both built using 35 cytokine predictive features. The best AUROC score of .99 with a 95% Confidence Interval (CI) (.982,.999) was achieved by the Random Forest classifier using 35 cytokine biomarkers. The second-best AUROC score of .954 with a 95% Confidence Interval (.929,.979) was achieved by the k-NN model using 35 cytokines. A *p*-value of less than 7.481e-10 obtained by an independent t-test validated that Random Forest classifier was significantly better than the k-NN classifier with regards to the AUROC score.

Presently, as large-scale efforts are gaining momentum to enable early, fast, reliable, affordable, and accessible detection of individuals at risk for CAD, the application of powerful ML algorithms can be leveraged as a supplement to conventional methods such as angiography. Early detection can be further improved by incorporating 65 novel and sensitive cytokine biomarkers. Investigation of the emerging role of cytokines in CAD can materially enhance the detection of risk and the discovery of mechanisms of disease that can lead to new therapeutic modalities.

## Background

### Introduction

Cardiovascular disease is the leading cause of death in Europe and North America [1, 2] which underscores the need for incorporation of novel emerging risk factors to improve prediction of risk, enabling early diagnosis and personalized management. The power of ML algorithms like k-NN and Random Forest can be harnessed to extract patterns to inform health related decision making. This paper expatiates the exploratory juxtaposition of k-NN and Random Forest by varying a broad spectrum of tuning parameters and incorporating k-fold cross validation, a powerful resampling technique that overcomes the issue of overfitting, ensuring better generalizability of the model [3]. Due to the limitations of data availability, before creating the model and final prediction, the data were augmented and balanced by Random Over-Sampling Examples technique (ROSE). ROSE, available from the Comprehensive R Archive Network (https://cran.r-project.org/web/Packages/ROSE/index.html) simulates balanced synthetic data by smoothed bootstrap approach.

We used 35 plasma cytokines as novel biomarkers to improve classification in patients with or without clinical coronary artery disease (CAD). This approach promises to identify mechanisms of disease with cytokine targets not previously recognized, and to improve early detection of individuals at risk. Cytokines are proteins generated by the immune system in response to cell signals. They act as messengers for other cells by targeted activation of receptors and trigger downstream, signaling. Common cytokines include lymphokines, chemokines, interferons, interleukins etc. that respond to environmental signals triggering pro- or anti-inflammatory cascades [4–6]. Cytokines are known to be involved in the development and progression of CAD [7].

### Review of related work

There are very few prior studies that have used ML algorithms to improve classification in patients with or without clinical coronary disease (CAD vs Controls) by using cytokines [5, 8] as predictive features, emphasizing the importance of the current study. The studies reviewed showcase how ML techniques to predict CAD versus Control can be used in conjunction with traditional methods or even independently to facilitate early, fast, affordable, accessible, noninvasive prediction without compromising accuracy. ML has gained impetus to offset the current limitations of the traditional methods utilized to diagnose CAD which are expensive and risky.

Alizadehsani, Roohallah, et al. [9] delineates the usage of ML algorithms in conjunction with angiography to obviate its disadvantages which include costs, complications, and after-effects of the invasive method. By the usage of ML algorithms, the researchers identified the subjects that were at a high risk and then these were referred for angiography. The study used laboratory and angiographic data to diagnose the degree of stenosis of each of the three main coronary arteries separately. Bagging and C4.5 classification algorithms were used to obtain an accuracy of 79.54,61.46 and 68.96% for diagnosis of stenoses of left anterior descending (LAD), left circumflex (LCX) and right coronary artery (RCA), respectively.

Mastoi, Qurat-ul-ain, et al. [10] investigated the automation of CAD diagnosis by the ML algorithms such as k-NN and SVM. The motive of the automation was to optimize the indication and avoid time consuming and expensive procedures that patients may undergo to diagnose CAD. The researchers developed an ML algorithm that was shown to be effective to optimize the indication of medically prescribed tests and procedures such as angiography, nuclear scan, and C-reactive protein, resulting in improved diagnostic accuracy, reduced cost and reduced risk for the patient. The features used for this classification were non-invasive tests and biomarkers such as electrocardiography (ECG), photoplethysmography (PPG), and phonocardiography (PCG). Other studies used clinical parameters such as age, blood pressure, and smoking habit. The best prediction accuracy of 99% was achieved by SVM.

Hampe, Nils, et al. [11] have proposed the exploration of cardiac computed tomography (CT) visualization by ML algorithms. CT generates detailed high spatial resolution with hundreds of slices which are under-utilized due to paucity of trained cardiac imagers and overwhelming workload for medical professionals. ML algorithms can surmount the obstacles of manual diagnosis by achieving accurate and fast results which might lead to additional secondary diagnosis as well. The survey explored and documented ML algorithms augmenting CAD detection and characterization spanning the past 10 years. The conclusion states that despite the challenges pertaining the implementation of ML within the clinical scope, the power of novel ML algorithms is providing an impetus to gain substantive insights in CAD classification.

Martin-Isla, Carlos, et al. [12] investigated the uses of ML algorithms for image-based diagnosis of CAD, which have accomplished deeper qualification and superior diagnosis due to the generative nature of ML algorithm that learn from past predictions. Furthermore, the potential of ML algorithm for CAD detection is emphasized by the extensive literature related to the domain.

Yu, Linghua, et al. [6] used cytokines to study inflammatory profiles in adult patients with studied hand, foot, and mouth disease (HFMD) prevalently found in the Asia-Pacific regions. The research participants implemented Random Forest to distinguish the HFMD disease group from the controls using 26 significant cytokines as predictor features. The findings of the research showcased correlation between enteroviral infection, genotype, and clinical presentation. The Random Forest algorithm achieved a final AUROC value of .91, demonstrating its excellent partition efficacy. This shows that cytokines are sensitive and powerful predictive biomarker features.

Struck, et al. [13] employed cytokine predictors, using Random Forest to differentiate malaria from a blood stream bacterial infection. The 7–15 cytokines used for the task were selected using ML classification techniques. The researchers used cytokines to offset the deficiency of a rapid malaria test not being able to differentiate serious malaria infection from asymptomatic malaria. This study exhibited a high disease status prediction accuracy of 88% that could provide directions to develop new point-of-care tests in Sub-Saharan Africa.

Saini, Indu, et al. [14] studied the usage of k-NN for the detection of QRS complexes in ECG related data. The authors showed that prediction accuracy primarily depended on the value of k and the distance metric used for classification. Running experiments, proved that Euclidean distance and a value of k = 3 in conjunction with 5-fold cross validation generated the best k-NN classifier. The prediction accuracy achieved was 99% which is remarkably high.

Ridker, Paul M, et al. [15] conducted CANTOS, a clinical trial funded by Novartis (Canakinumab Anti-inflammatory Thrombosis Outcome Study) whose objective was to investigate the involvement of interleukin-1 β in the inflammation at a cellular level. The experiment entailed giving the men in the study a monoclonal antibody against interleukin-1 β. The anti-inflammatory therapy targeted the interleukin-1 β innate immunity pathway with canakinumab at a dose of 150 mg every 3 months. The study exhibited a significant lower rate of cardiovascular events than placebo independent of lipid lowering.

The current study endeavored to improve and extend the techniques in the published studies. The primary objective is to leverage the powerful algorithms like k-NN and Random Forest with the emerging cytokine biomarkers to obtain a better separability evaluated by the AUROC curve.

## Methods

The data set is composed of biomarker levels for 104 individuals. Thirty-five cytokine biomarkers were measured for all in addition to the final target feature attribute which assigns individuals to the CAD (39 individuals) or the Control (65 individuals) group. The feature space in the model incorporates 35 cytokine biomarkers to quantify the similarity and finally the classification of CAD or Control.

This study was approved by the UCSF Institutional Review Board Committee on Human Research and conducted in accordance with the principles of the Declaration of Helsinki. All subjects provided written informed consent prior to participation. For this study, blood samples were collected from male (43.3%) and female subjects, ages 18 to 65 (median age = 42) with diagnosed CAD, and from age, and sex-matched controls. CAD subjects had a previous history of myocardial infarction, angiographically diagnosed CAD, or previous coronary artery bypass graft surgery. Control subjects had no history or clinical evidence of CAD. Exclusion criteria included current or prior treatment for autoimmune disease and/or cancer, diabetes, tobacco use, NSAID use prior to blood collection, post-menopausal women, and age over 65. None of the subjects were receiving lipid lowering medications. Blood was drawn into EDTA collection tubes and immediately stored on ice. Samples were centrifuged to separate plasma which was aliquoted and stored at − 80 °C until use. Samples were thawed on ice and assayed for cytokine content with a human 35-plex ELISA assay (ThermoFisher/Life Sciences) according to the manufacturer protocol. The raw data were analyzed by xPonent software and were expressed in pg/mL using the standard curve for each cytokine. Table 1 describes the clinical demographic profile of the subjects in the study.

**Table 1:** Clinical Demographic Profile. A collection of plasma samples from patients with diagnosed coronary artery disease (CAD) and healthy controls

Gender	CAD (***n*** = 39)	Control (***n*** = 65)	Total (***n*** = 104)
**Male**	19	26	45 (43.27%)
**Female**	20	39	59 (56.73%)
**Total**	39 (37.5%)	65 (62.5%)	104

### Pre-processing steps

Prior to running the classifier on the testing set, the attributes/features of the data were normalized to prevent the predictive features with larger values from dominating the features having smaller values which would result in a biased classification. Additionally, the data were checked and remediated for discrepancies such as null values or outlier values by consulting with domain experts. Due to the availability of a small data set which was significantly imbalanced, the data were incremented synthetically by smoothened bootstrap mechanism which prevented overfitting in training phase and translated in better generalizability during the testing phase. By using the ROSE Package from the R programming language, the data were synthetically increased to a size of 1000 and balanced with CAD accounting for 52% of the cases and Controls 48% of the cases. The ROSE Package helps achieve this by simulating smoothened bootstrap approach. The data was scaled by z-score standardization.

The final balanced data composed of 52% CAD and 48% Control is reasonable for implementing k-NN and Random Forest. The augmentation of the data to 1000 observations in conjunction with data balancing allows for a conventional 75–25% split toward the training and testing partitions. This segmentation will allow for enough observations to be included within the training set to avoid underfitting the model. Additionally, 10-fold cross validation repeated three times was implemented for choosing hyperparameters to prevent biased predictions. The overall predictive feature space of 35 cytokines was used because after balancing and augmenting data, feature selection was not required to avoid underfitting or overfitting the model. The optimal balance of bias variance (underfitting versus overfitting) tradeoff was achieved by these strategies.

### k-NN K-nearest neighbor

k-NN, a supervised ML algorithm was initially proposed by Fix and Hodges [16]. It is based upon the similarity paradigm, indicating that the classification of unlabeled examples is differentiated by means of distance metrics and are finally ascribed the class of k (k ≥ 1) nearest neighbors. k-NN, by being non-parametric in nature does not make assumptions regarding the underlying data distribution, therefore making it less restrictive and a more powerful classifier as compared to other popular ML algorithms. k-NN is a versatile algorithm that can be used for classification as well as prediction via Regression.

*k*-NN is a lazy learner therefore does not create a learning model, but instead every new testing instance is iterated through the training data to decide upon its class label. An increase of data instances causes a higher computational complexity due to the lack of the abstraction phase. k-NN also has the disadvantage that it does not predict well for data that are noisy or have outliers. Despite the caveats, the availability of computational power in contemporary context as well as the hyper-parameters that can be tuned for k-NN, allow it to be leveraged to adequately classify testing examples in a reasonable amount of time.

In the past as well as in the present, a plethora of complex medical research has applied k-NN [17] to achieve optimal diagnostic prediction. k-NN is prevalently used for detecting genetic diseases, conducting facial recognition, and generating music recommendations. The choice of this algorithm stems from the fact that even though classification can be slow, k-NN is fundamentally a simplistic algorithm which typically uses numeric predictor features, is easily comprehendible and outperforms many of the more complex ML algorithms.

## Parameter K (number of neighbors) fine tuning

The optimal value of hyper parameter k is decided by empirically initiating the algorithm with k = 1 and iteratively incrementing k until the classifier’s error rate is minimized. This technique helps prevent under fitting as well as overfitting of the testing data thereby balancing the bias variance trade-off. If k is too small, there is a reasonable possibility that an outlier will affect the classification and if k is too large the similarity neighborhood might incorporate several deviant classes. For a noisy dataset, where the nearest neighbors vary widely in their distances, closest neighbors are more reliable for class label characterization and are given priority weighting by the process of majority vote.

## Similarity distance metric parameter

To compute the k-NN similarity index using the contextual feature space, the distance between two feature vectors is prevalently measured using Euclidean distance. Much of the current analysis was implemented using the R Package caret [18], (available from the Comprehensive R Archive Network at http://CRAN.R-project.org/Package=caret) and R Package ggplot2 [19], (available from the Comprehensive R Archive Network at https://cran.r-project.org/web/Packages/ggplot2/index.html).

The Euclidean distance was implemented for the current research which is derived for a L_2_-norm.

### Minkowski distance

Mathematically, the distance d(x, y) in a D-dimensional feature space between two points.

x = (x_1_, x_2_, x_3_……x_D_)^T^ and y = (y_1_, y_2_, y_3_……y_D_)^T^ is represented as follows:

1 $$ \mathbf{d}\left(\mathbf{x},\mathbf{y}\right)={\left\Vert \mathbf{x}-\mathbf{y}\right\Vert}_{\mathbf{p}}={\left({\sum}_{\mathbf{i}}^{\mathbf{D}}{\left|{\mathbf{x}}_{\mathbf{i}}-{\mathbf{y}}_{\mathbf{i}}\right|}^{\mathbf{p}}\right)}^{\mathbf{1}/\mathbf{p}} $$

The L_p_-norm is defined as the Minkowski distance where p is the factor depicting the norm.

### Euclidean distance

If *p* = 2, L_2_-norm is defined as the Euclidean distance.

2 $$ \mathbf{d}\left(\mathbf{x},\mathbf{y}\right)={\left\Vert \mathbf{x}-\mathbf{y}\right\Vert}_{\mathbf{2}}={\left({\sum}_{\mathbf{i}}^{\mathbf{D}}{\left|{\mathbf{x}}_{\mathbf{i}}-{\mathbf{y}}_{\mathbf{i}}\right|}^{\mathbf{2}}\right)}^{\mathbf{1}/\mathbf{2}} $$

### Random Forest

The Random Forest classification model consists of many decision trees operating as an ensemble, that result in the target class with the majority vote assigned to the test example. The low correlation between models helps to ensure that the composite classification of the ensemble outperforms any individual classification by offsetting the errors of each model. Bagging or Bootstrap Aggregation are used to implement diversity within the tree models. Each model uses randomly sampled training data extracted with replacement that generates a distinct tree. This procedure does not allow replicating the training data since sub-setting a record cannot be chosen more than once. Additionally, unlike a simple decision tree, the ensemble decision trees are forced to split on a node as per a randomly selected distinct feature, which might not be the best partition criterion resulting in low correlation amongst the differentiated parallel trees. The trees in the Random Forest are not only disparate regarding training data, but also regarding node split feature partition.

### Optimization techniques

The optimizing mechanism of k-fold cross validation as well as inclusion of statistically significant cytokines were incorporated to enhance the final classification result.

### k-fold cross validation

k-fold Cross Validation is a technique that optimizes the prediction ability of a model in the context of new unlabeled data consequently offsetting issues like overfitting or selection bias. The technique entails partitioning a dataset into k complementary subsets, implementing training of the model on k-1 subsets, and finally validating it on one partition. This study used k = 10 to implement 10-fold cross validation, repeated 3 times.

### Classifier experimental framework

Across both classifiers repeated 10-fold cross validation, data scaling and balancing was implemented by using the R caret Package. The first classifier experiment entailed applying the k-NN algorithm involving 35 cytokine predictor features with the Euclidean distance. The second classifier implemented the Random Forest with 35 cytokines.

**Fig. 1:**
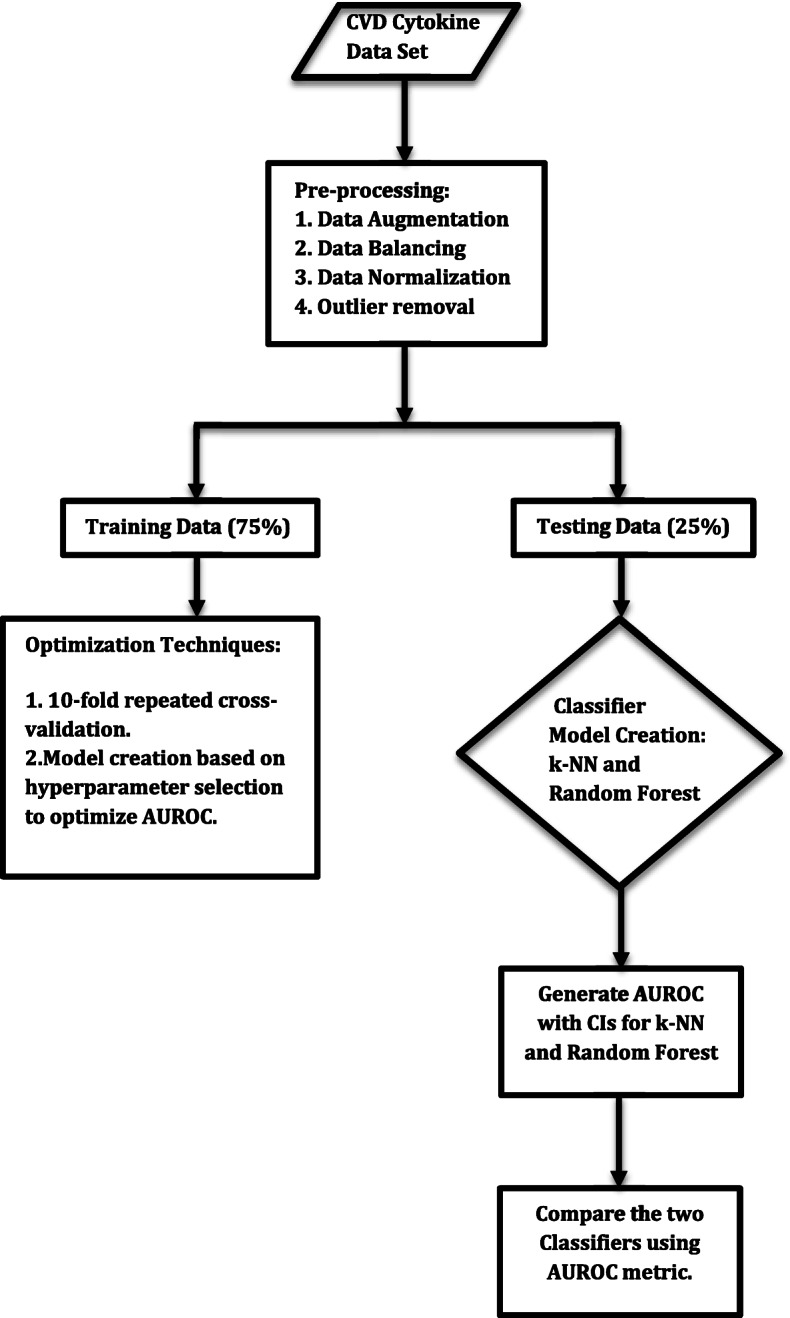
The classifier framework implemented to identify “At Risk” Coronary Artery Disease (CAD) classification

The graphical representation of the Classifier Experimental framework is provided in the following figure (Fig. 1).

### Evaluation measures

A versatile set of performance evaluation measures are available to obtain insights related to the efficacy of algorithms in terms of accuracy. For this study we used AUROC to obtain the accuracy of differentiating the CAD versus Control groups and for comparing the performance of the classifiers implemented.

### AUROC (area under receiver operating characteristic)

This is a standard measure that helps determine the degree of separability achieved by the relevant Classification algorithm. Higher AUROC showcases the algorithm’s capability of accurately differentiating the instances into the target classes. The AUROC curve is created by plotting False Positive Rate (FPR) on the x-axis against the True Positive Rate (TPR) on the y-axis. AUROC is a better comparative measure of accuracy across distinct classifiers from the standpoint of statistical consistency and discrimination.

## Results

### Testing results

**Fig. 2:**
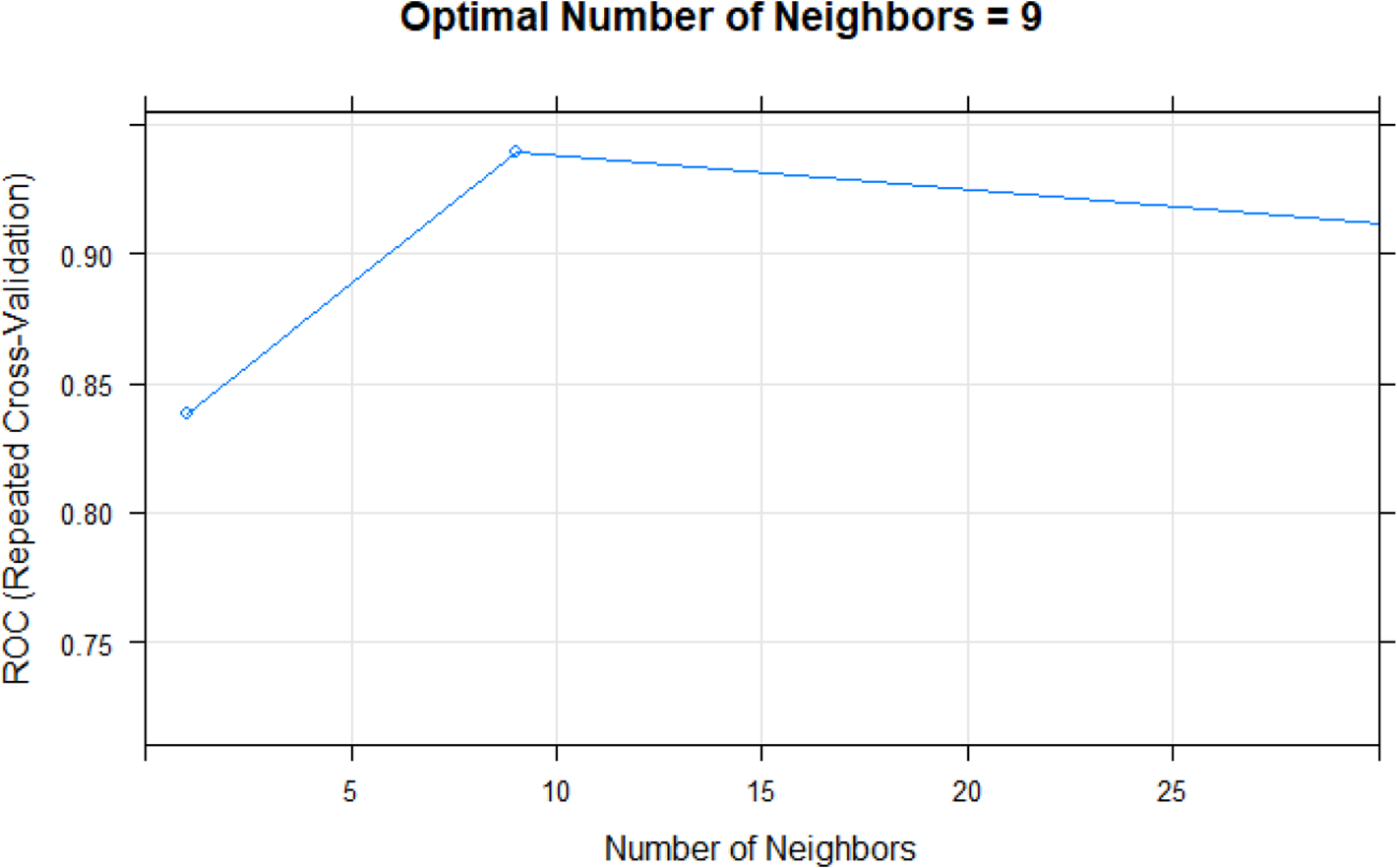
Classifier 1 Experiment: Optimal number of neighbors for k-NN (Euclidean Distance) with a total set of 35 cytokines

**Fig. 3:**
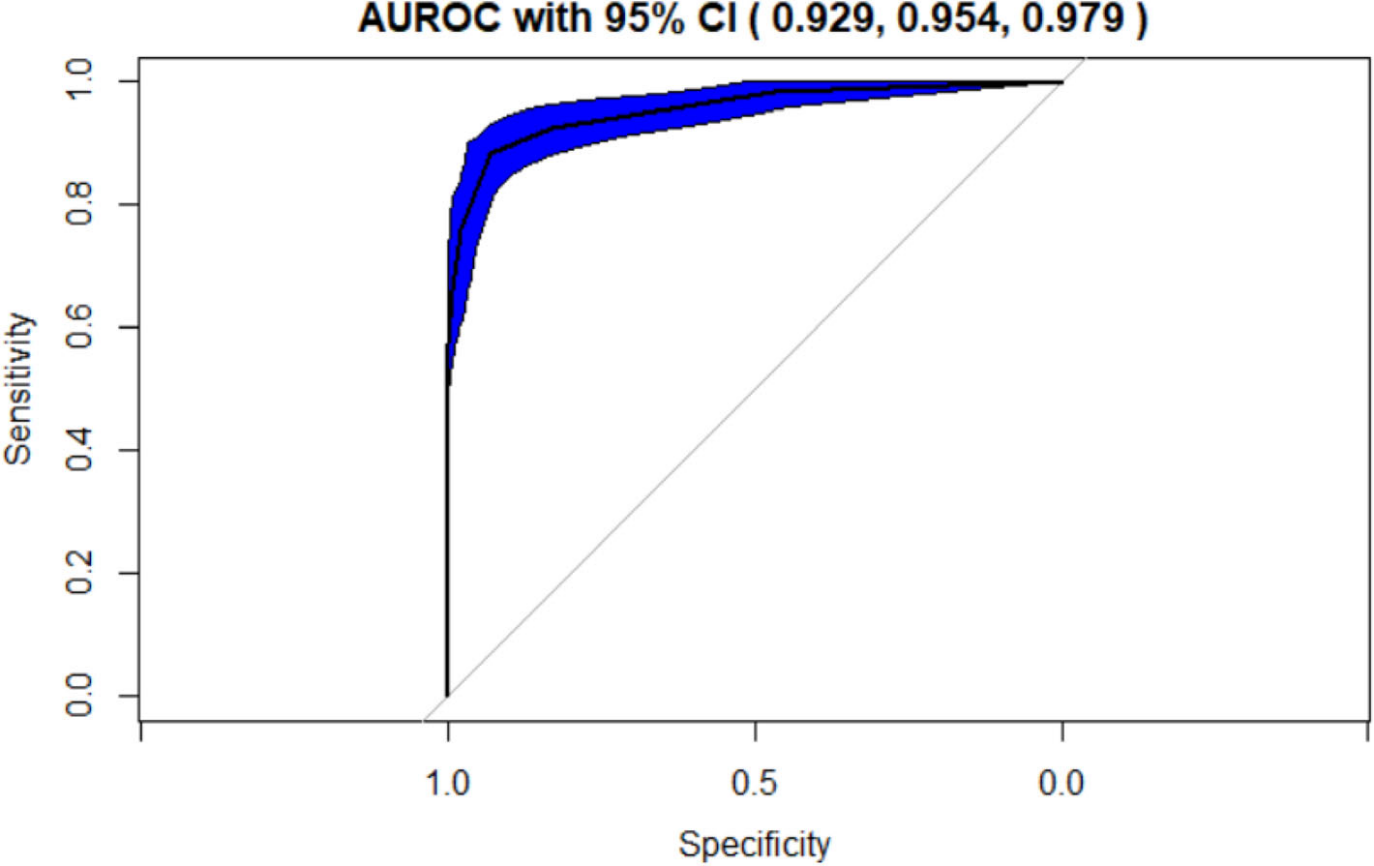
Classifier 1 Experiment: AUROC curve with 95% CI for k-NN (Euclidean Distance) with a total set of 35 cytokines

**Fig. 4:**
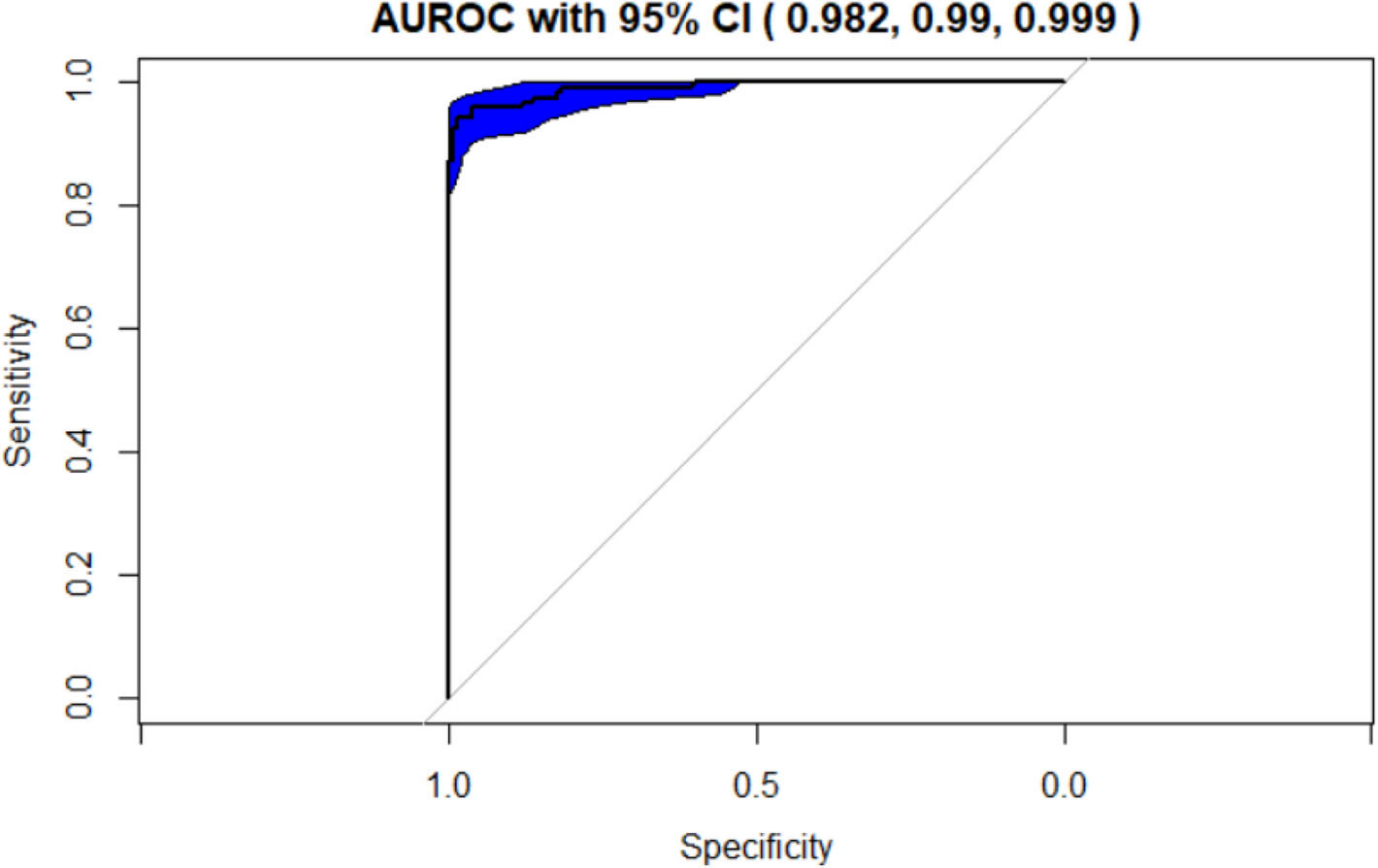
Classifier 5 Experiment: AUROC curve for Random Forest with the total set of 35 cytokines

The testing results obtained by running the algorithm on the test data are displayed in the following tables (Tables 2 and 3) and graphs (Figs. 2, 3 and 4). The testing results applied the hyperparameter tuning metrics optimized by the resampling repeated 10-fold cross validation results.

**Table 2:** Classifier 1 Experiment Results for the k-NN algorithm with 35 cytokines and k = 9

Algorithm	Classification Criterion	Predictor Feature Space	AUROC with 95% Confidence Interval	Prediction Accuracy	Sensitivity	Specificity
k-NN	Distance Measure: Euclidean with k = 9	35 Cytokines	**0.954 (.929, .979)**	0.832	0.992	0.658

**Table 3:** Classifier 2 Experiment Results for the Random Forest with 35 cytokines

Algorithm	Classification Criterion	Predictor Feature Space	AUROC with 95% Confidence Interval	Prediction Accuracy	Sensitivity	Specificity
Random Forest	Decision Trees	35 Cytokines	**0.99 (.982, .999)**	0.96	0.954	0.967

#### Classifier 1 experiment

This classifier used k-NN with Euclidean distance measure and k = 9 to classify the “At Risk” instances. For this classification 35 cytokines were used. The AUROC value of .95, representing the extent of separability of CAD versus Control was remarkable. The details related to AUROC, optimal k-NN neighbor ascertained via cross-validation and numeric measures for Classifier 1 are provided in the table and graph above **(**Table 2, Figs. 2 and 3)**.**

#### Classifier 2 experiment

The Classifier 2 implemented Random Forest using 35 cytokines as predictor features. The AUROC of .99 was extremely high and surpassed the accuracy achieved by the previous classifier. This accomplishment can be accounted for by the underpinnings of the Random Forest algorithm. Five hundred decision trees, 6 feature variables as split criterion, gini index as quality evaluator were used as hyperparameters for the Random forest classification. The processes of random feature split criteria, creation of multiple decision trees and cumulating the intermediary results from these trees results in the observed outstanding performance. The diverse trees used in the decision-making process bolster the accuracy and stability of the final prediction. The final numeric metric results and AUROC for Classifier 2 are listed in the table and graph above **(**Table 3, Fig. 4).

#### Comparing classifiers

**Fig. 5:**
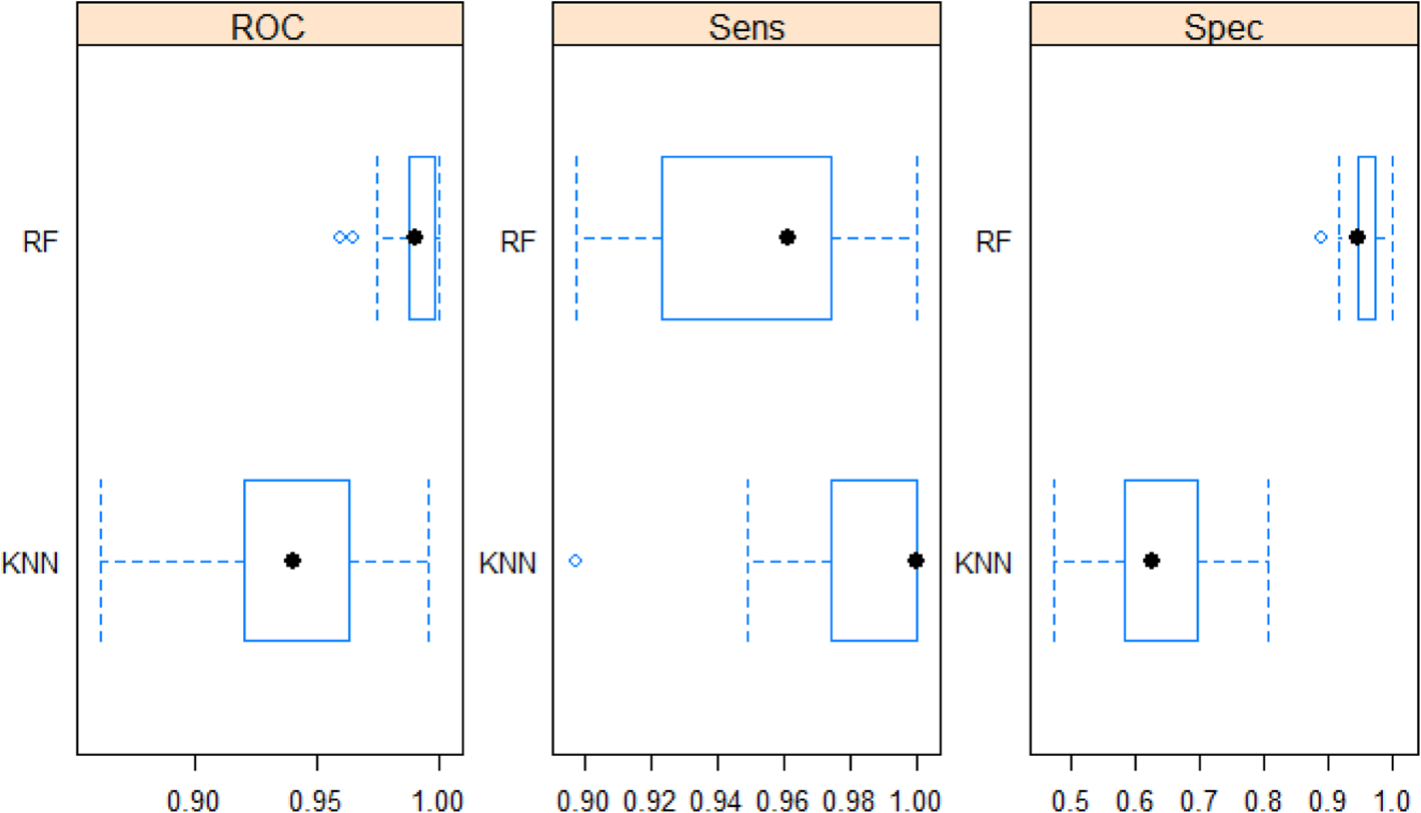
Classifier Comparison: AUROC curve, Sensitivity, Specificity for Random Forest and k-NN with the total set of 35 cytokines

The algorithm can be compared by Confidence Intervals in conjunction with t tests to check for significant difference with regards to AUROC values. Visually the two classifiers can be compared by boxplots. The independent t test comparison proved that the AUROC values for the two classifiers were significantly different with a *p*-value less than 7.481e-10. The pair wise boxplot comparisons with regards to AUROC, Sensitivity and Specificity are displayed in the following graph (Fig. 5):

## Discussion

Random Forest with 35 cytokines (**Classifier 2 Experiment**) overall proved to be the best ML classification technique as demonstrated by the AUROC score of **0.99** with a **95% CI (.982, .999).** The Random Forest outperformed the other model and demonstrated an almost perfect AUROC because it is composed of an ensemble of uncorrelated decision tree models that collectively provide a better classification compared to that generated by individual models. Random Forest, like bagging, creates trees from bootstrap samples. Additionally, Random Forest selects a subset of features at each partition that creates trees. This is a desirable characteristic resulting in diversity within the tree predictions and uncorrelated prediction errors. A random subset of features is used at each split point to create trees and this diversifies the ensemble therefore enabling better overall performance than algorithms like k-NN.

k-NN with 35 cytokines (**Classifier 1 Experiment**) provided an AUROC value of **0.954** with a **95%** **CI (.929, .979)**. This is a highly effective classifier though not as good as the Classifier 2 which provided a nearly perfect CAD versus Control differentiation.

The t test (***p*-value < 7.481e-10**) comparing the AUROC score for the two classifiers exhibited that the Random Forest Classifier (Classifier 2) is significantly better than the k-NN Classifier (Classifier 1).

## Conclusions

The ubiquitous implementation of ML algorithm has gained momentum because of the availability of high computational power and the outstanding prediction accuracy which is an improvement of the current qualitative assessment of images and crude quantitative measures of cardiac structure and function. The ML algorithm can build a holistic framework encompassing not just images but also other informative features to obtain credible insights and early detection which will result in saving lives.

The studies which were reviewed, and the current research here underscore the importance of ML techniques and how they can be harnessed to predict patients at high risk for CAD to inform traditional methods to direct treatment. k-NN, a similarity metric-based algorithm and Random Forest [3], an algorithm based on an ensemble of trees are popular and have been used across a broad spectrum of domain areas including medical diagnosis of which some prominent ones are discussed here.

The current study exhibited an exceptional prediction accuracy which is a significant improvement as compared to the one showcased by Alizadehsani, Roohallah, et al. [9]. Random Forest, implemented in this study achieved the highest AUROC (.99) score which was better than the reviewed study conducted by Yu, Linghua, et al. [6]

This prediction mechanism is not a substitute for the conventional methods such as angiography but can be used to inform the recommendations for more advanced tests in patients at risk with more serious disease. The usage and comparison of multiple algorithms has proven an effective way to obtain a holistic view with regards to the classification. The current study incorporates this comparison paradigm to present the [20] results. The empirical proof of enhanced performance using hyperparameter tuning and cross fold validation [21] for Random Forest and k-NN directed the in-depth exploration conducted in the current research. Fine tuning hyperparameters in general has proven to be an effective optimization technique. The novel cytokine biomarkers that are indicators of inflammation [15, 20, 22] can be leveraged to make the identification of CAD risk more substantive and could be comprehensive targets for future therapies.

This research uniquely implements the use of cytokine plasma biomarkers to differentiate CAD from Control cases. Additionally, it emphasizes the exploratory paradigm of multiple classifier experiments that show improved prediction accuracy across different models. The k-NN algorithm implementation were compared in terms of efficacy as well as juxtaposed relative to the performance of Random Forest algorithm. As compared to prior research studies [6, 13] that used Random Forest with cytokines to differentiate disease groups from controls, our study exhibited better AUROC **(.99**).

Both Random Forest and k-NN generated reasonably good results with all the 35 cytokines. For both k-NN and Random Forest classifier experiments were balanced for the bias variance trade off by performing cross-validation, data augmentation data balancing and using the 75–25% split towards training and testing set. Overall, Random Forest provided superior AUROC of **0.99** with **95% CI (.982, .999)**, prediction accuracy of **0.96** metrics and was proved to be significantly better than k-NN classifier by the independent t test with a *p*-value of less than **7.481e-10**.

In this age of innovation and all-pervasive ML systems, it is important to leverage the abstraction, generalization, optimization, and computational power of the versatile ML algorithms that can be used across a wide spectrum of domains of which medical sciences is a very prominent one. Future investigative research of the role of cytokine profiles to identify inflammation suffered by CAD subjects will translate to therapeutic targets.

Contemporary research indicates that numerous biological factors contribute to risk of CAD including individual molecular species of lipoproteins, oxidative stress, and genetic determinants of inflammation and coagulopathy. The analytical mathematical techniques emerging from this research will permit the analysis of a much broader array of factors, including their mutual interaction in the appreciation of risk of CAD. The inclusion of a broad array of cytokines will contribute a new dimension to this analysis that can lead to improved risk prediction and novel therapeutic interventions.

## Data Availability

The data used for this research comprises confidential patient health information. The data obtained from the Kane laboratory and Genomic Resource in Cardiovascular and Metabolic Disease at UCSF are HIPPA protected and cannot be released publicly. The data, without personal identification, are delineated within the manuscript and we are prepared to answer any questions that researchers might have regarding the data usage for the experimental framework.

## Abbreviations

AUROC: Area under Receiver Operating Characteristic
CAD: Coronary Artery Disease
CI: Confidence Interval
FPR: False Positive Rate
k-NN: K Nearest Neighbor
ML: Machine Learning
ROSE: Random Over-sampling Examples
TPR: True Positive Rate
